# Oncogenic *GNAQ* mutations are not correlated with disease-free survival in uveal melanoma

**DOI:** 10.1038/sj.bjc.6605226

**Published:** 2009-08-04

**Authors:** J Bauer, E Kilic, J Vaarwater, B C Bastian, C Garbe, A de Klein

**Affiliations:** 1 Department of Dermatology, University of Tübingen Medical Center, Liebermeisterstr. 25, Tübingen 72076, Germany; 2 Department of Ophthalmology, Erasmus MC, University Medical Center, Rotterdam, The Netherlands; 3 Departments of Dermatology and Pathology, and Comprehensive Cancer Center of University of California at San Francisco, San Francisco, CA, USA; 4 Department of Clinical Genetics, Erasmus MC, University Medical Center, Rotterdam, The Netherlands

**Keywords:** uveal melanoma, survival, oncogenic mutation

## Abstract

**Background:**

Recently, oncogenic G protein alpha subunit q (*GNAQ*) mutations have been described in about 50% of uveal melanomas and in the blue nevi of the skin.

**Methods:**

*GNAQ* exon 5 was amplified from 75 ciliary body and choroidal melanoma DNAs and sequenced directly. *GNAQ* mutation status was correlated with disease-free survival (DFS), as well as other clinical and histopathological factors, and with chromosomal variations detected by FISH and CGH.

**Results:**

Of the 75 tumour DNA samples analysed, 40 (53.3%) harboured oncogenic mutations in *GNAQ* codon 209. Univariate and multivariate analysis showed that *GNAQ* mutation status was not significantly correlated with DFS.

**Conclusion:**

The *GNAQ* mutation status is not suitable to predict DFS. However, the high frequency of *GNAQ* mutations may render it a promising target for therapeutic intervention.

Cutaneous melanocytic nevi and melanomas show frequent oncogenic mutations in *BRAF* and *NRAS*, and consequent constitutive activation of the MAP-kinase pathway (Davies *et al*, 2002; Pollock *et al*, 2003). In contrast, melanocytic tumours such as uveal melanomas rarely show *BRAF* and *NRAS* mutations (Kilic *et al*, 2004; Saldanha *et al*, 2004; Janssen *et al*, 2008; Maat *et al*, 2008). Recently, somatic mutations in the heterotrimeric G protein alpha subunit q (*GNAQ*) have been found to be associated with dominant dark skin in a large-scale mutagenesis screen of inbred C3HeB/FeJ mice (Van Raamsdonk *et al*, 2004). In humans, *GNAQ* was found to be frequently mutated in the blue nevi of the skin (83%) and uveal melanoma (46%) (Van Raamsdonk *et al*, 2009). The mutations occur exclusively in codon 209 in the ras-like domain and result in constitutive activation, turning *GNAQ* into a dominant acting oncogene, activating the MAP-kinase pathway. We asked whether oncogenic *GNAQ* mutations in uveal melanoma are associated with patient survival.

## Materials and methods

### Patients and tumour material

Ciliary body or choroidal melanomas were collected from patients who underwent enucleation of the tumour-containing eye at the Erasmus MC Rotterdam or Rotterdam Eye Hospital (Rotterdam, The Netherlands). Informed consent was obtained before enucleation and the study was performed according to the tenets of the Declaration of Helsinki. Fresh tumour material was obtained within 1 h after enucleation and processed for FISH and cytogenetic analysis as described (Kilic *et al*, 2005). A fraction of each tumour was snap-frozen and stored in liquid nitrogen. The remainder of the eye was embedded in paraffin and sections were stained with haematoxylin and eosin (H&E) for evaluation. All tumours were confirmed histopathologically as uveal melanomas (on the basis of H&E morphology, mitotic activity, and results of HMB45 and S100 staining). Seventy-five patients were selected from our extensive database with information on follow-up and clinical, cytogenetic and histopathological parameters.

### FISH and CGH analysis

Dual-colour FISH on uncultured tumour material using centromeric and locus-specific cosmid, P1 or YAC probes for chromosome 1, 3, 6 and 8 was performed as described previously (Kilic *et al*, 2005). CGH analysis was performed on DNA from formalin-fixed paraffin-embedded tumour material as described previously (Kilic *et al*, 2005).

### *GNAQ* mutation analysis

*GNAQ* exon 5, which includes the mutational hotspot codon 209, was amplified from uveal melanoma biopsy DNA using PCR and the primers 5′-CCCACACCCTACTTTCTATCATTTAC-3′ and 5′-TTTTCCCTAAGTTTGTAAGTAGTGC-3′. PCR products were purified using ExoSAP-IT (USB, Staufen, Germany), and sequenced in reverse direction directly on an ABI Prism 3700 DNA Analyzer (Applied Biosystems, Foster City, CA, USA).

### Statistical analysis

The primary end point for disease-free survival (DFS) was the time to development of metastatic disease; death due to other causes was treated as censored. Statistical analyses were performed with SPSS software, release 16.0 (Munich, Germany). Actuarial probabilities of DFS (with an event defined as development of metastatic disease or death by disease) were estimated according to the Kaplan–Meier method. To examine the possibility that other clinical, histopathological or chromosomal variations affected the prognosis, we performed Cox proportional hazard analysis for each confounding variable. An effect was considered significant if the *P*-value was ⩽0.05.

## Results

### Tumour pathology and clinical outcome

A total of 75 uveal melanomas were included in the study. The median age of the patients at the time of enucleation was 62 years (range 21–86). In all, 39 patients were female and 36 male. Based on their cell type 14 tumours were classified as epithelioid, 28 as mixed, and 33 as spindle cell type. The mean tumour diameter and thickness were 13.4 mm (range 7.0–19.0) and 8.4 mm (range 1.5–20.0), respectively. At the end of the follow-up (mean 56.1 and range 6.4–136.4 months), 28 patients died of melanoma-related disease, 1 patient was diagnosed with metastases, 9 patients died due to other causes and 37 patients were still alive without metastases.

### Molecular genetic analysis

All uveal melanomas were analysed for the oncogenic *GNAQ* mutation and chromosomal changes of chromosomes 3 and 8. Of the 75 tumour DNA samples analysed, 40 (53.3%) harboured oncogenic mutations in *GNAQ* codon 209. In detail, 29 cases showed a heterozygous Q209P mutation, 1 case a homozygous Q209P mutation, 9 cases a heterozygous Q209L and 1 case a Q209R mutation. The copy number of chromosome 3 was obtained in 74 uveal melanomas, and of chromosomal region 8q in 68 uveal melanomas.

### Statistical analysis

**Figure 1:**
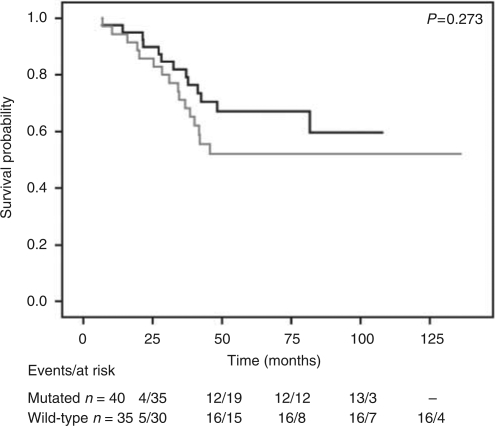
Kaplan–Meier survival curve of tumours harbouring mutated *vs* wild-type GNAQ. The black line represents mutated, and the grey line represents wild type. The table shows the number of events and cases at risk over time at the respective time points.

**Figure 2:**
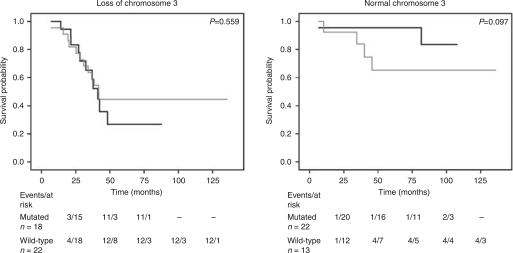
Kaplan–Meier survival curve of *GNAQ* mutations *vs* wild type stratified for loss of chromosome 3. Black line represents mutated GNAQ, and the grey line represents wild type. The table shows the number of events and cases at risk over time at the respective time points.

Univariate analysis was performed for all parameters, showing a lower DFS for patients with loss of chromosome 3 and gain of chromosome 8q. Univariate analysis of *GNAQ* mutated cases compared with wild-type tumours did not show a significantly decreased DFS (*P*=0.273) (Figure 1). To examine the possibility that *GNAQ* mutations may affect the prognosis of patients with loss of one copy of chromosome 3, we calculated Kaplan–Meier survival curves of *GNAQ* status stratified for chromosome 3 status and performed log rank tests (*P*=0.559) (Figure 2). Disease-free survival was not modified by the presence of *GNAQ* mutations. In tumours with two copies of chromosome 3 the patients with a *GNAQ* mutation seemed to have a better prognosis, although it was not significant (*P*=0.097). Correlations between the clinical, chromosomal parameters and *GNAQ* mutation were calculated using Mann–Whitney and Fisher's exact tests (Table 1). We did not observe any significant correlations. When analysed in a multivariate model with *GNAQ* as a confounding variable, we obtained a hazard ratio of 1.07 with a *P*-value of 0.854 (data not shown).

## Discussion

In a cohort of 75 uveal melanoma patients, we could show that DFS was not significantly correlated with *GNAQ* mutation status. However, analysis stratified for loss of chromosome 3 as well as multivariate analysis was clearly limited by the small sample size, and hence the results should be interpreted with caution.

The mutation frequency of *GNAQ* codon 209 (53%) was in the same range as that in a recent report by Onken *et al* (2008) (49%) and by Van Raamsdonk *et al* (2004) (46%), confirming the importance of oncogenic *GNAQ* mutations in uveal melanoma. However, *GNAQ* mutations have been shown to have similar frequencies at all clinical stages of uveal melanoma progression, and to be independent of chromosomal aberrations, hinting at *GNAQ* being an early or initiating oncogenic event (Onken *et al*, 2008). This is consistent with the assumption that frequent oncogenic mutations of *BRAF* and *NRAS* in cutaneous melanoma as well as in benign melanocytic nevi (Davies *et al*, 2002; Pollock *et al*, 2003), which also activate the MAP-kinase pathway, are early events and are not associated with clinical outcome (Shinozaki *et al*, 2004; Akslen *et al*, 2005; Edlundh-Rose *et al*, 2006).

In conclusion, we could show that oncogenic *GNAQ* mutations are not suitable to predict the clinical outcome in uveal melanoma. However, the high frequency of *GNAQ* mutations may render it a promising target for therapeutic intervention.

## Figures and Tables

**Table 1:** Correlation between *GNAQ* mutation and chromosomal abnormalities and clinical data

	***GNAQ* mutation status**		
	**Mutated**	**Wild type**	***P*-value**
*Clinical data*			
Gender			
Male	17	19	0.555
Female	18	21	
Mean age (years)	64.5	60.5	0.220
Cell type			
Spindle	15	18	0.519
Mixed/epithelioid	20	22	
Mean tumour diameter	12.9	13.8	0.205
Mean tumour thickness	8.3	8.5	0.842
Involvement of the ciliary body			
Yes	6	5	0.404
No	29	35	
			
*Chromosomal abnormalities*			
Chromosome 3 loss			
Yes	22	18	0.094
No	13	22	
Chromosome 8q gain			
Yes	22	21	0.100
No	8	17	

*GNAQ*=G protein alpha subunit q.
